# Regulatory network features in *Listeria monocytogenes*—changing the way we talk

**DOI:** 10.3389/fcimb.2014.00014

**Published:** 2014-02-14

**Authors:** Veronica Guariglia-Oropeza, Renato H. Orsi, Haiyuan Yu, Kathryn J. Boor, Martin Wiedmann, Claudia Guldimann

**Affiliations:** ^1^Department of Food Science, Cornell UniversityIthaca, NY, USA; ^2^Department of Biological Statistics and Computational Biology, Cornell UniversityIthaca, NY, USA; ^3^Department of Biological Statistics and Computational Biology, Weill Institute for Cell and Molecular Biology, Cornell UniversityIthaca, NY, USA

**Keywords:** *Listeria monocytogenes*, regulatory network, PrfA, SigB, network motif

## Abstract

Our understanding of how pathogens shape their gene expression profiles in response to environmental changes is ever growing. Advances in Bioinformatics have made it possible to model complex systems and integrate data from variable sources into one large regulatory network. In these analyses, regulatory networks are typically broken down into regulatory motifs such as feed-forward loops (FFL) or auto-regulatory feedbacks, which serves to simplify the structure, while the functional implications of different regulatory motifs allow to make informed assumptions about the function of a specific regulatory pathway. Here we review the basic concepts of network features and use this language to break down the regulatory networks that govern the interactions between the main regulators of stress response, virulence, and transmission in *Listeria monocytogenes*. We point out the advantage that taking a “systems approach” could have for our understanding of gene functions, the detection of distant regulatory inputs, interspecies comparisons, and co-expression.

## Introduction

Genetic studies used to be the main approach of studying regulatory mechanisms. These studies usually analyze small, closed regulatory systems, involving no more than four or five regulators and often only analyze a single regulatory mechanism involving a regulator and its regulatee. Advances in Bioinformatics have made it possible to model complex systems, including increasingly large regulatory networks in bacteria (Dufour and Donohue, 2012; van Helden et al., 2012), higher organisms (Middleton et al., 2012; Klinger et al., 2013), and chemical systems (Kamerlin et al., 2011, recently recognized with a Nobel Prize in Chemistry). The integrated analysis of all known regulatory interactions in an organism via a systems approach (Barabasi and Oltvai, 2004; Snoep et al., 2006) into one large network is possible, with data as diverse as RNA-seq, ChIP-seq, and microarray data as inputs (for a practical example see Bonneau et al., 2007). In these analyses, regulatory networks are typically broken down into regulatory motifs such as feed-forward loops (FFL) or autoregulatory feedbacks. Breaking down a network into these “building blocks” serves to simplify the structure, while the functional implications of different regulatory motifs allow the generation of informed assumptions about the function of a specific regulatory pathway. There are a number of examples on how network studies have been harnessed to discover new functions for known transcription factors (Bonneau et al., 2007), protein-protein interactions (Wichadakul et al., 2009), assign functions to genes with unknown functions (Bonneau et al., 2007), compare the same network across species and study their evolution and diversification (Wuchty et al., 2003), and compare different networks in the same organism (Xia et al., 2004; Yu and Gerstein, 2006).

The aim of this mini review is to give an overview of basic network motifs and their function, to use this framework to identify and explore different regulatory network motifs, and explore key regulatory networks in *Listeria monocytogenes (L. monocytogenes)*. This approach will illustrate the benefits of taking a systems approach to a comprehensive analysis of large networks in *L. monocytogenes* and other bacterial pathogens.

In order to survive, bacteria must adapt to their environment, and to do so they express an array of regulatory factors responsible for mounting a specific and rapid response to changes in their surroundings. The foodborne pathogen *L. monocytogenes* has the ability to adapt to diverse conditions encountered in the extra-host environment (e.g., soil, food), the gastro-intestinal tract, and the extra- and intracellular environment encountered in different hosts. In order to overcome these changing conditions, *L. monocytogenes* expresses an arsenal of effector proteins encoded by genes that are tightly regulated by alternative σ (sigma) factors, transcriptional activators, transcriptional repressors and at the translational and post-translational levels. While transcriptional regulators are known to activate/repress a set of genes in response to a stimulus, in many cases the response is not as straightforward and adaptation to a particular stress often involves a network of regulators that can interact directly, or indirectly, through an activation cascade and/or coregulation.

In *L. monocytogenes*, several regulators involved in the control of gene expression have been identified and characterized in detail. The positive regulatory factor A (PrfA) regulates the expression of the vast majority of virulence genes (Scortti et al., 2007), therefore its own expression is tightly regulated at the transcriptional, translational and post-translational levels (reviewed in de las Heras et al., 2011) The concentration of PrfA and its affinity for the promoter will ultimately determine the strength of the PrfA response. This balance is achieved through the combination of different mechanisms such as basal transcriptional control (Chaturongakul et al., 2008), autoregulatory transcription loops (Scortti et al., 2007), and a translational thermoswitch that represses activation outside the host (Johansson et al., 2002) amongst others (reviewed in Freitag et al., 2009).

Another major input of regulation at the transcriptional level is achieved through the action of sigma factors, the promoter recognition subunits of RNA polymerase holoenzyme. In *L. monocytogenes*, σ^B^regulates the expression of general stress response genes and therefore plays a crucial role in the survival of this bacterium in challenging environments (reviewed in O'Byrne and Karatzas, 2008). Besides σ^B^, the *L. monocytogenes* genome encodes for two (in lineages I, II and IV isolates) to three (in lineage II isolates) additional alternative σ factors. σ^L^ regulates approximately 20 genes (Arous et al., 2004) and has been shown to be involved in low temperature resistance, salt and lactic acid stress (Chan et al., 2008; Raimann et al., 2009; Tessema et al., 2012). σ^H^ regulates approximately 50 genes (Chaturongakul et al., 2011) and appears to be involved in alkaline stress (Rea et al., 2004) and σ^C^, an extra cytoplasmic σ factor specific to lineage II strains, has been shown to be activated by heat stress (Zhang et al., 2005). Other regulators involved in *L. monocytogenes* stress response include CtsR and HrcA, two negative regulators involved in heat shock stress (Nair et al., 2000). CodY is a nutrient responsive regulator with a possible role in mediating response to temperature stress (Bennett et al., 2007) and AgrA is a temperature dependent, autoregulatory protein involved in virulence (Autret et al., 2003; Garmyn et al., 2012). Additionally, over 15 two-component systems have been reported in *L. monocytogenes*, several of them involved in response to different stresses (Glaser et al., 2001; Williams et al., 2005; Chan et al., 2008).

Increasing evidence supports that there are many ways in which regulators interact to fine-tune *L. monocytogenes* gene expression in response to different environmental conditions. For example, overlaps in the regulons of PrfA, CtsR, HrcA, and σ^B^, σ^C^, σ^H^, and σ^L^ have been shown (Chaturongakul et al., 2011). Similarly, the AgrA regulon has been shown to overlap with the PrfA, σ^B^, σ^H^, and CodY regulons (Garmyn et al., 2012). A number of more specific interactions between regulators have also been defined. For example, both CodY and σ^B^ have been shown to be involved in regulation of PrfA expression (Ollinger et al., 2009; Lobel et al., 2012). Additionally, increasing data is available on the role of non-coding RNA that interfere with gene regulation at the transcriptional, translational and post-translational level (reviewed in Mellin and Cossart, 2012). Overall, existing data support complex regulatory networks that allow *L. monocytogenes* to fine-tune its response of to the rapidly changing conditions and to integrate diverse stimuli to regulate specific phenotypic responses. Further studies of these networks are needed to understand their function under different conditions with a higher level of detail and resolution.

## Network features—an overview of concepts

**Figure 1:**
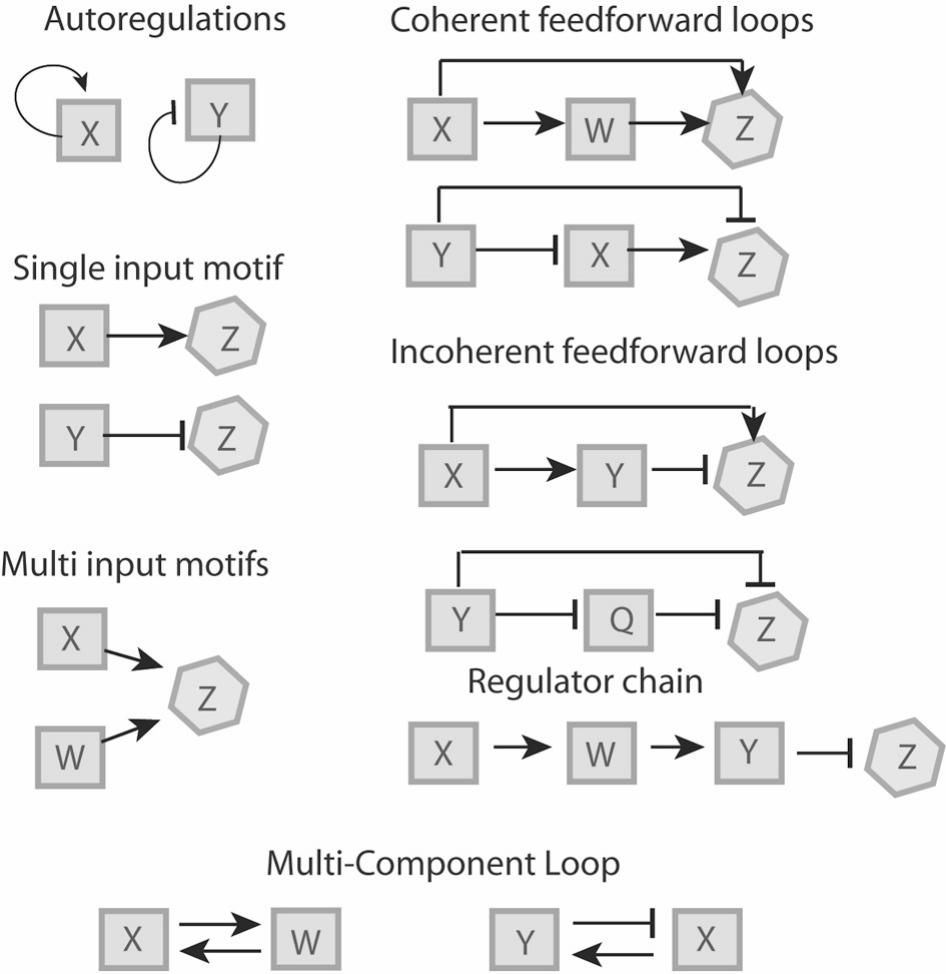
**Regulatory features**. Proteins “X” and “W” are positive regulators, proteins “Y” and “Q” are negative regulators and protein “Z” is a non-regulator. All six motifs represented can be recognized in *L. monocytogenes* (see Figure 2).

Mathematical modeling allows for the identification of commonly used regulatory elements, or network motifs, which can be used as building blocks to understand larger network structures (reviewed in Alon, 2007; Tyson and Novak, 2010). These elements can be broken down into six motifs (Figure 1) which will be described below. These motifs have been described for bacteria (Shen-Orr et al., 2002) and yeast (Lee et al., 2002), some of them are more common and overrepresented whereas others are rare. In general, network motifs offer the possibility to study complicated regulatory systems on a higher level of abstraction.

### Single input motif

Under a specific condition, a positive (X) or negative (Y) transcription factor binds to a specific set of genes (operon Z), which are solely regulated by either X or Y. Functionally, single input motifs facilitate a fast and straightforward response, for example in reaction to a specific condition (Shen-Orr et al., 2002).

### Multi-input motif

A set of transcriptional factors X and W are able to recognize and bind to the same promoter region of a set of genes (operon Z). A multi-input motif allows the coordination of gene expression in response to different signals (Shen-Orr et al., 2002).

### Feed-forward loop

A transcriptional factor X regulates transcriptional factor W and both of them directly regulate a set of genes, operon Z. In a coherent FFL, both regulators have the same effect on Z (e.g., X activates W and both X and W activate Z). The coherent FFL serves as a signal-sensitive delay element that can be dose- or time-dependent (Mangan et al., 2003). In an incoherent FFL, both regulators have antagonistic roles on Z (e.g., X activates Y and Z, but Y represses Z). Incoherent FFL have been studied in detail, and they are thought to provide a biphasic behavior where phase one involves a rapid activation with a concomitant phase two of delayed inhibition (Mangan and Alon, 2003; Kim et al., 2008). These loops often serve to minimize noise, i.e., fluctuations in gene expression, therefore fine-tuning their regulatory response (Thattai and van Oudenaarden, 2001). In pathogens, these loops may allow for activation of specific genes that are required only in specific compartments, followed by rapid downregulation to prevent expression in a subsequent compartment where expression of a given protein may be detrimental.

### Autoregulation

A transcriptional factor recognizes the promoter of its own gene. There are conceivable advantages of auto-regulation, such as fast reaction to stimuli and low biosynthetic cost of regulation (McAdams and Arkin, 1997; Thieffry et al., 1998; Becskei and Serrano, 2000; Guelzim et al., 2002; Lee et al., 2002; Shen-Orr et al., 2002).

### Multi-component loop

Two or more regulatory factors are involved in a closed circuit. While multi-component loops have been described in yeast, they were initially thought to be absent in bacteria (Lee et al., 2002). However, recent studies have shown a few rare examples of multi-component loops in bacterial genetic networks (Ruiz et al., 2001; Kato et al., 2003).

### Regulator chain

Three or more regulators involved in the sequential activation of each other. Time-dependent events such as the cell cycle and developmental features such as spore formation often involve regulator chains (de Hoon et al., 2010).

### Regulatory network features in *L. monocytogenes*

In this section, we apply the above principles to analyze regulatory network features in *L. monocytogenes*, focusing on the different interactions between σ^B^ and PrfA and their contribution to transcription and translation of genes with roles in virulence and stress response.

### Single input motif

**Figure 2:**
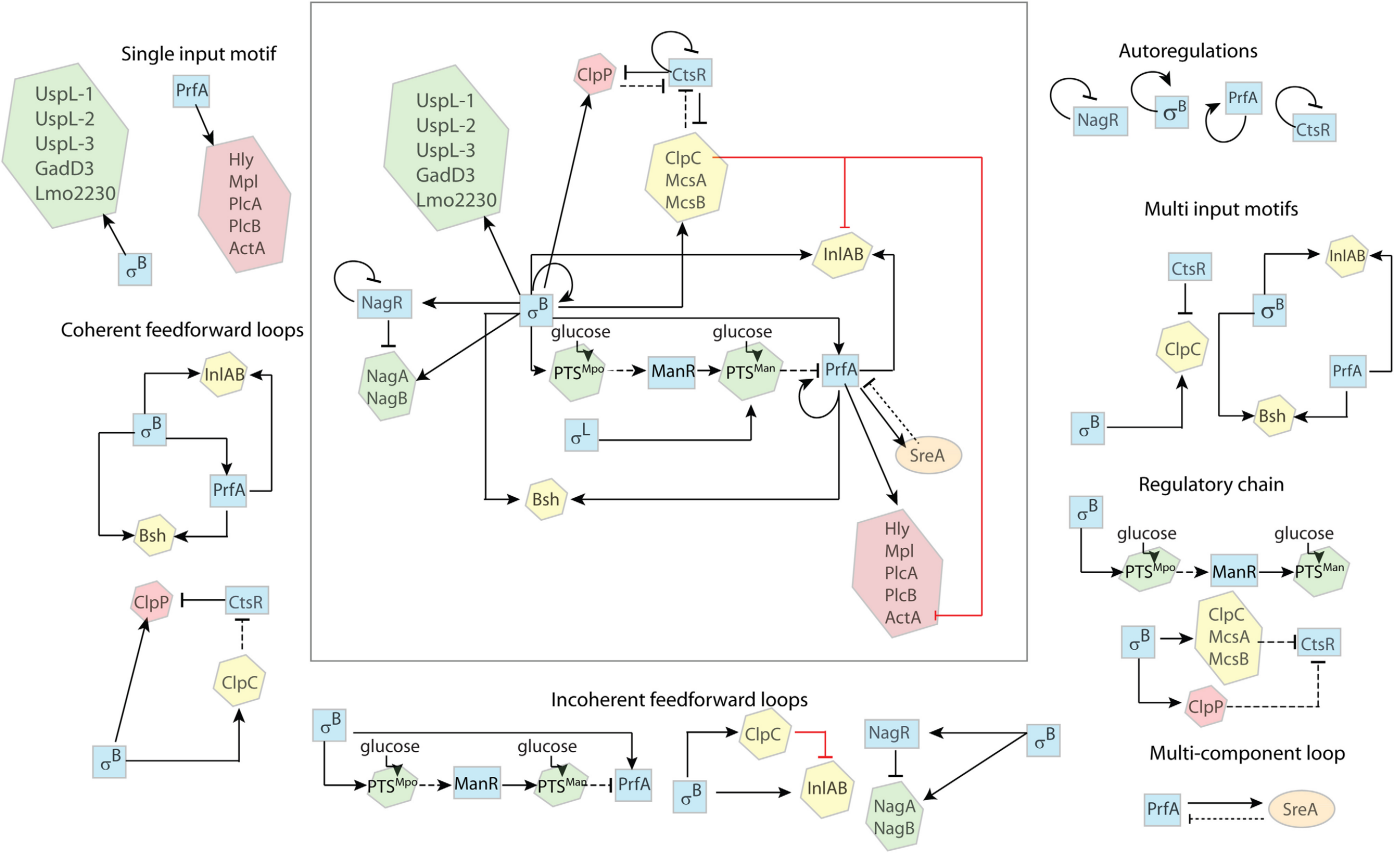
**Regulatory features in *L. monocytogenes***. The regulatory network involves six transcriptional regulators; the alternative σ factors σ^B^ and σ^L^, the transcription activators PrfA and ManR, and the transcription repressors CtsR and NagR (blue squares). Proteins not involved in transcription regulation are enclosed in hexagons. Proteins mostly active during environmental growth (green) include NagA, an N-acetyl-glucosamine-6-phosphate deacetylase, NagB, a glucosamine-6-phosphate deaminase, PTS^*Mpo*^ and PTS^*Man*^, two glucose PTSs; ClpC, a protease, InlA and InlB, two internalin proteins, and Bsh, a bile salt hydrolase, are involved in the early stages of infection (yellow); ClpP is a serine protease and Hly, Mpl, PlcA, PlcB, and ActA are virulence factors involved in the intracellular stage of infection (red). SreA is a trans-acting noncoding RNA. Solid arrows (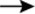) indicate activation and crossed lines (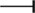) indicate repression. Solid black lines indicate transcriptional regulation (i.e., regulation at the DNA level); dotted black lines indicate post-transcriptional regulation (i.e., regulation at the RNA level); dashed black lines indicate post-translational regulation (i.e., regulation at the protein level); red solid lines indicates unknown mechanism of regulation (i.e., transcriptional, post-transcriptional, or post-translational). The complex regulatory system is broken down into simpler regulatory features as described in the text.

Examples of single input motifs are genes that are solely regulated by σ^B^ (e.g., *uspL*-1, *uspL*-2, *uspL*-3 Seifart et al., 2011, *lmo2230* Kazmierczak et al., 2003; Utratna et al., 2012, *gadD3* Wemekamp-Kamphuis et al., 2004; Oliver et al., 2009). While transcriptional regulation by PrfA of the core virulence genes *plcA*, *hly*, *mpl*, *actA*, *plcB* may also be viewed as a single input regulatory motif (Figure 2), transcription of these genes requires both σ^A^ as well as PrfA and thus should probably be considered a multi input motif. While some may not consider σ^A^ a regulator as it is the constitutively active housekeeping sigma factor, σ^A^ levels may still change, which would at least show minor effects on gene regulation.

### Multi input motif

Overlapping regulons are common in bacteria and there are several examples in *L. monocytogenes* where multiple regulators regulate the same genes (Chaturongakul et al., 2011). For example, σ^B^ and PrfA co-regulate transcription of at least three genes, including the *inlAB* operon and *bsh* (Figure 2). Both *inlAB* and *bsh* have independent and distinct PrfA and σ^B^ dependent promoters (Lingnau et al., 1995; Dussurget et al., 2002; Kazmierczak et al., 2003). CtsR and σ^B^ also co-regulate transcription of the *mcsA-mscB-clpC* operon, which includes a σ^B^ dependent promoter (upstream of *mcsA*) and an independent σ^A^ dependent promoter with a CtsR binding site (upstream of *ctsR* itself) (Hu et al., 2007a).

### Positive autoregulation

PrfA, the main positive regulatory factor of virulence genes, can upregulate its own transcription (Figure 2). PrfA can be transcribed as a monocistronic mRNA from the two promoters directly upstream of the PrfA gene, p1prfA which is σ^A^ dependent and p2prfA which is σ^B^ and σ^A^ dependent (de las Heras et al., 2011). PrfA can also be transcribed as a bicistronic mRNA from the PrfA-dependent promoter upstream of *plcA* (Mengaud et al., 1991; Scortti et al., 2007), creating a positive autoregulatory feedback loop.

Another example of autoregulation is the transcription of *sigB*, which occurs from a σ^B^-dependent promoter upstream of the *L. monocytogenes rsbVW-sigB-rsbX* operon (Kazmierczak et al., 2003) (Figure 2).

### Negative autoregulation

Examples for negative autoregulation in *L. monocytogenes* include regulation of CtsR and HrcA, both of which are negative regulators of heat shock proteins (Hu et al., 2007b) (Figure 2). Both CtsR (Nair et al., 2000) and HcrA (Hanawa et al., 2000) can bind to their own promoter and repress transcription of the *ctsR-mcsA-mcsB-clpC* and *hcrA-grpA-dnaK* operons respectively.

### Coherent feedforward loops

*L. monocytogenes* has several coherent FFL that involve both σ^B^ and PrfA, including transcription of *bsh* and *inlAB*. *bsh* encodes a bile salt hydrolase, which contributes to the bacterial defense against bile salts (Dussurget et al., 2002; Jones et al., 2008). *inlA* encodes internalin A (InlA), a bacterial surface molecule that mediates the entry of *L. monocytogenes* into mammalian epithelial cells (Lingnau et al., 1995). The coherent FFL for both *bsh* and *inlAB* involves (i) direct transcriptional activation of these genes by σ^B^ and (ii) σ^B^-dependent transcription of PrfA (Schwab et al., 2005), which in turn directly activates both *bsh* (Dussurget et al., 2002; Kazmierczak et al., 2003; Sue et al., 2003, 2004) and *inlAB* (McGann et al., 2007) transcription (Figure 2).

An example for a coherent FFL that works via inhibitory mechanisms is the regulation of ClpP, a heat shock protein that is involved in intracellular growth (Gaillot et al., 2000, 2001). This coherent FFL involves (i) upregulation of *clpP* transcript levels by σ^B^, possibly through a putative σ^B^-dependent promoter upstream of *clpP* (Wemekamp-Kamphuis et al., 2004) and (ii) σ^B^-dependent transcription of the *mcsA*-*mcsB*-*clpC* operon (Gaillot et al., 2001) with ClpC, McsA, and McsB mediated post-translational inhibition of CtsR (Chaturongakul and Boor, 2006; Hu et al., 2007a), which relieves CtsR mediated transcriptional downregulation of *clpP* (Chaturongakul et al., 2011), resulting in increased ClpP levels (Figure 2). It is conceivable that this FFL serves to increase the level of ClpP under the acidic conditions encountered during gastrointestinal passage (via indication of the acid responsive σ^B^), therefore priming the bacteria for more efficient subsequent intracellular growth.

### Incoherent feedforward loops

One example of an incoherent FFL is represented by σ^B^-dependent regulation of *inlAB*, which includes (i) positive transcriptional regulation of *inlAB* through σ^B^ (Kazmierczak et al., 2003) and (ii) indirect σ^B^-dependent downregulation of *inlAB* expression, which involves σ^B^ activating the expression of ClpC (Hu et al., 2007a; Chaturongakul et al., 2011), which has been shown to downregulate, through an unknown mechanism, the transcription of *inlA* and *inlB* (Nair et al., 2000) (Figure 2).

A potentially very important example of an incoherent FFL can be found in the interaction between σ^B^ and PrfA. There is a σ^B^-dependent direct upregulation of *prfA* transcription as well as a σ^B^-dependent indirect post-translational inhibition of PrfA (Nadon et al., 2002; Ollinger et al., 2009). This incoherent FFL may facilitate rapid activation of PrfA, with a subsequent delayed inhibition of PrfA-dependent gene regulation to moderate the negative effects of prolonged activation of PrfA-dependent genes such as *hly* (Scortti et al., 2007), which may cause host cell lysis when overexpressed. This incoherent FFL includes (i) activation of *prfA* transcription via the σ^B^-dependent p2*prfA* promoter (Nadon et al., 2002) and (ii) σ^B^-dependent downregulation via a regulatory chain that involves *mpoABCD* (encoding PTS^Mpo^), *manR* (encoding ManR), *manLMN* (encoding PTS^Man^), and *prfA* (encoding PrfA), as described in detail below (Figure 2). Ollinger et al. (2009) initially reported evidence for σ^B^-dependent downregulation of PrfA by an unknown mechanism that did not involve downregulation of *prfA* transcription. Recently, Ake et al. (2011) showed that the the σ^A^- and σ^B^-induced *mpo* operon, which encodes the PTS complex PTS^Mpo^, is involved in inactivation of PrfA through a cascade of post-translational and transcriptional regulation, providing a potential mechanism for σ^B^-dependent downregulation of PrfA. This regulation involves PTS^Mpo^ itself, ManR, a transcriptional activator of the *man* operon, the PTS^Man^ complex and PrfA (Raengpradub et al., 2008; Oliver et al., 2009, 2010; Ollinger et al., 2009; Tessema et al., 2009; Mujahid et al., 2013a). In the proposed model, upon uptake of glucose through PTS^Mpo^, two subunits of PTS^Mpo^ become dephosphorylated and, then, prevent the inhibitory phosphorylation of ManR. The functional ManR then activates the transcription of the *manLMN* operon, which encodes for PTS^Man^. Upon uptake of glucose by PTS^Man^, the EIIAB^Man^ subunit becomes dephosphorylated, which inhibits PrfA by a mechanism not yet elucidated (Dalet et al., 2001; Arous et al., 2004; de las Heras et al., 2011; Mujahid et al., 2013b).

Incoherent FFL are functionally suited for the regulation of metabolic enzymes. Energy conservation warrants the transcription of catabolic enzymes only when the substrate is present. Therefore, the repressor of the respective enzymes is often co-translated in the same operon or at least under the control of the same transcription factor. The presence of the appropriate substrate then inactivates the repressor. An example for this is part of the chitin catabolism of *L. monocytogenes*. The chitin monomer GlcNAc is an ubiquitous source of carbon and nitrogen. It is used by many microorganisms (Resch et al., 2010) and can be exploited by bacteria in a dual way: it is either degraded into fructose-6-P and funneled into glycolysis for energy production, or it can be used anabolically in peptidoglycan synthesis (Bertram et al., 2011; Popowska et al., 2012). In *L. monocytogenes,* GlcNAc degradation is regulated by an incoherent FFL with (i) σ^B^ positively regulating transcription of genes that facilitate GlcNAC degradation (*nagA* and *nagB*) and (ii) σ^B^ positively regulating NagR, which negatively regulates transcription of *nagA* and *nagB* (Figure 2). Briefly, σ^B^ upregulates transcription of the *nagABR* operon (Raengpradub et al., 2008; Mujahid et al., 2013a). NagA and NagB are GlcNAc metabolic enzymes, and NagR is a transcriptional repressor that inhibits the transcription of *nagABR* unless the substrate (GlcNAc) for NagA and NagB is present. NagA (N-acetylglucosamine-6-phosphate deacetylase) and NagB (glucosamine-6-phosphate deaminase) facilitate the degradation of GlcNAc into fructose-6-P (Popowska et al., 2012).

### Multi component loop

An example of a multi component loop in *L. monocytogenes* is the recently shown regulation involving (i) PrfA positively regulating *sreA* and (ii) SreA negatively regulating PrfA. SreA is a S-adenosylmethionine (SAM) riboswitch, and *sreA* transcription has been shown to be PrfA-dependent with a 7-fold increase during intracellular growth (Loh et al., 2009). Moreover, it has been shown that, in addition to controlling the expression of downstream genes, the SreA riboswitch also functions as a small noncoding RNA, acting post-transcriptionally to decrease the expression of PrfA (Loh et al., 2009; Mellin and Cossart, 2012).

### Regulator chain

Two examples of regulator chains in *L. monocytogenes* are (i) the σ^B^-dependent repression of CtsR and (ii) the σ^B^-dependent activation of PTS^Man^ (see Figure 2). Briefly, the σ^B^-dependent repression of CtsR involves σ^B^-mediated transcriptional upregulation of McsA, McsB, ClpC, and ClpP (Hu et al., 2007a; Chaturongakul et al., 2011) and, as observed in *B. subtilis* (Kruger et al., 2001), subsequent degradation of CtsR by the ClpCP protease along with McsA and McsB. Similarly, σ^B^ also upregulates the transcription of *mpoABCD* (Raengpradub et al., 2008; Oliver et al., 2009, 2010; Ollinger et al., 2009; Mujahid et al., 2013b), which encodes PTS^Mpo^. Upon glucose uptake by PTS^Mpo^, one subunit, EIIB^Mpo^, post-translationally activates the transcriptional regulator ManR, which then, activates the transcription of the *manLMN* operon (encoding PTS^Man^) (Ake et al., 2011).

### Closing remarks

A holistic systems approach to regulatory networks will be essential to provide new insights into gene regulation in *L. monocytogenes*. Studying complex regulatory interactions in motifs enables the detection of distant connections more easily as it shortens pathways into motifs without specifically naming all the intermediary steps. The abstraction to regulatory motifs also makes comparison across different species easier, since motif analysis will detect similarities in the hardwiring of a network regardless of the names of individual factors. Another important application of regulatory motifs in the analysis of regulatory networks is the study of co-expression. If two genes are co-regulated by the same transcription factor the degree of co-expression may vary (Yu et al., 2003). Experimental determination of co-expression for one motif will allow for informed assumptions about the degree of co-expression in a similarly wired regulatory network. However, one of the current challenges is the often missing information of the precise biological function of a network. Experimental confirmation of assumptions made from network analyses remains crucial.

As new genetics and “omics” data involving regulatory interactions in *L. monocytogenes* become available, the need to develop better tools to analyze these interactions on a large scale grows and a systems approach to understanding regulatory networks becomes feasible.

A better understanding of how transcriptional regulators affect the expression of downstream regulatees is key to understanding the biology of *L. monocytogenes* and other bacterial pathogens that have to transit rapidly changing environments to cause disease and will ultimately facilitate the development of better strategies to prevent and treat listeriosis.

### Conflict of interest statement

The authors declare that the research was conducted in the absence of any commercial or financial relationships that could be construed as a potential conflict of interest.
